# Unravelling the Belgian cascade of hypertension care and its determinants: insights from a cross-sectional analysis

**DOI:** 10.1186/s12889-024-19010-x

**Published:** 2024-06-14

**Authors:** Philippe Bos, Edwin Wouters, Katrien Danhieux, Josefien van Olmen, Roy Remmen, Kerstin Klipstein-Grobusch, Daniel Boateng, Veerle Buffel

**Affiliations:** 1 Department of Sociology, University of Antwerp, Antwerp, Belgium; 2 Department of Family Medicine and Population Health, University of Antwerp, Wilrijk, Belgium; 3 Julius Global Health, Department of Global Health and Bioethics, Julius Center for Health Sciences and Primary Care, University Medical Center Utrecht, Utrecht University, Utrecht, The Netherlands; 4 Department of Sociology, Vrije Universiteit Brussel, Brussels, Belgium

**Keywords:** Hypertension, Blood pressure, Cascade of care, Prevalence, Awareness, Treatment, Control, Risk factors, Socioeconomic determinants

## Abstract

**Background:**

Hypertension is a major risk factor for cardiovascular disease and all-cause mortality worldwide. Despite the widespread availability of effective antihypertensives, blood pressure (BP) control rates remain suboptimal, even in high-income countries such as Belgium. In this study, we used a cascade of care approach to identify where most patients are lost along the continuum of hypertension care in Belgium, and to assess the main risk factors for attrition at various stages of hypertension management.

**Methods:**

Using cross-sectional data from the 2018 Belgian Health Interview Survey and the Belgian Health Examination Survey, we estimated hypertension prevalence among the Belgian population aged 40–79 years, and the proportion that was (1) screened, (2) diagnosed, (3) linked to care, (4) in treatment, (5) followed up and (6) well-controlled. Cox regression models were estimated to identify individual risk factors for being unlinked to hypertension care, untreated and not followed up appropriately.

**Results:**

The prevalence of hypertension based on self-reported and measured high BP was 43.3%. While 98% of the hypertensive population had their BP measured in the past 5 years, only 56.7% were diagnosed. Furthermore, 53.4% were linked to care, 49.8% were in treatment and 43.4% received adequate follow-up. Less than a quarter (23.5%) achieved BP control. Among those diagnosed with hypertension, males, those of younger age, without comorbidities, and smokers, were more likely to be unlinked to care. Once in care, younger age, lower BMI, financial hardship, and psychological distress were associated with a higher risk of being untreated. Finally, among those treated for hypertension, females, those of younger age, and without comorbidities were more likely to receive no adequate follow-up.

**Conclusion:**

Our results show that undiagnosed hypertension is the most significant barrier to BP control in Belgium. Health interventions are thus needed to improve the accurate and timely diagnosis of hypertension. Once diagnosed, the Belgian health system retains patients fairly well along the continuum of hypertension care, yet targeted health interventions to improve hypertension management for high-risk groups remain necessary, especially with regard to improving treatment rates.

**Supplementary Information:**

The online version contains supplementary material available at 10.1186/s12889-024-19010-x.

## Background

Raised blood pressure (BP) is identified as a major risk factor for cardiovascular disease (CVD) [1] and all-cause mortality, accounting for an estimated 8.5–10.8 million annual deaths worldwide [2, 3]. Despite the decreasing age-standardized prevalence of high BP in high-income countries (HICs) over the last decades [2] and substantial improvements in hypertension awareness, treatment and control [4, 5], the proportion of people that are well controlled in most HICs remain far below those that are — and therefore can be — achieved in high-quality hypertension programs [4]. It is estimated that in high-income Western countries, in 2019, only 37% of hypertensive men and 43% of hypertensive women had their BP under control [5]. Estimated hypertension control rates in Belgium — the country of this study — were even lower, at 34.7% for men and 42.3% for women [5].

Thus, despite the wide availability of antihypertensive drugs and their well-established efficacy in lowering BP [6], control rates in Belgium remain suboptimal. The reason is that hypertension is a chronic condition, requiring lifelong coordinated and continuous action from healthcare systems along the continuum of care [7]. Hypertensive patients need to be identified, diagnosed, initiated to treatment and adequately followed up in order to achieve long-term adherence to therapy and the resulting health outcomes.

Previous research on hypertension control in Belgium is scarce and the few studies that were conducted show varying BP control rates ranging between 21.5 and 45% [8–12]. These studies are, however, either outdated [11, 12] or have been limited to hypertensive individuals that are already in treatment [8–10]. In order to better understand the suboptimal control rates, there is a need to broaden the focus of attention and to identify where and why patients are lost along the entire care trajectory. Uncontrolled hypertension may, for instance, result from a lack of screening or failure to initiate treatment after a diagnosis.

In addition, especially groups with lower socio-economic status often do not receive the appropriate chronic care and support [13]. This may be particularly the case in Belgium: although the overall strength of the Belgian primary care system is considered as strong, it performs only weakly in terms of accessibility [14, 15], with levels of unmet medical need for the lower income quantiles ranging among the highest in Europe [16, 17]. This raises the need to evaluate the delivery of hypertension care using a Cascade of Care (CoC) approach and to identify vulnerable groups that have an elevated risk for attrition from hypertension care.

The CoC is a heuristic tool that visualizes patient retention across the sequential steps of chronic illness management and allows to quantify and identify the points of greatest attrition [18, 19] — thereby broadening the perspective to also include hypertensive individuals that are not in treatment. While originating from studies as early as the 1960s to evaluate the success of tuberculosis programs [20], the CoC framework gained widespread popularity in the evaluation of the delivery of HIV care [19]. Due to its promising results, it has subsequently been applied to other chronic diseases, such as diabetes [21] and hypertension [7, 22–25].

The aim of the current study is twofold. First, to identify the points of greatest attrition along the continuum of hypertension care in Belgium, and hence where health interventions are most necessary. Second, to identify patients’ characteristics that are associated with an elevated risk of attrition from hypertension care. By doing so, we hope to provide valuable insights for policymakers to develop targeted health interventions that address the unique needs of these high-risk groups, ultimately improving BP control.

## Methods

### Data sources

This study uses cross-sectional data from the sixth Belgian Health Interview Survey (BHIS) [26] and the first Belgian Health Examination Survey (BHES) [27], both conducted in 2018. The BHIS is a recurring cross-sectional survey (at four-to-five-year intervals) that collects information on the health status, health behaviour and health consumption of a representative sample of the Belgian population, through a combination of face-to-face interviews and self-administered questionnaires. Respondents were selected from the national population register using a stratified multi-stage cluster sampling procedure [26, 28].

The BHES was organized as a second stage of the BHIS and collects additional health information through clinical examinations and analyses of blood and urine samples among a subsample of the BHIS respondents [27]. The BHES contains, for instance, data on BP readings, allowing a reliable estimation of the prevalence of hypertension (including the diagnosed and non-diagnosed). During the BHIS interviews, all respondents at least 18 years of age, excluding proxy respondents and residents of the German-speaking community, were recruited to partake in the BHES. Recruitment continued until predefined regional quotas were achieved, which totalled a sample size of 1100 respondents [27]. A detailed elaboration on the methodology of both surveys is described elsewhere [26–28].

In line with prior research [4] and the target age group of the general physicians’ guidelines for the management of hypertension in Belgium [29, 30], the current analysis has been restricted to individuals aged 40–79 years. This resulted in an analytic sample of 5932 and 813 respondents for the BHIS and BHES, respectively. Both data sources were linked using a unique identifier code, so that for the BHES sample both the BHIS and BHES data were available.

## Measures and definitions

### The cascade of hypertension care

To construct the cascade of hypertension care, we distinguish between 7 sequential stages: (1) prevalence, (2) screening, (3) diagnosis, (4) linkage to care, (5) treatment, (6) follow-up, and (7) BP control.

To estimate the *prevalence*, hypertension was defined as either having a systolic BP (SBP) ≥ 140 mmHg or a diastolic BP (DBP) ≥ 90 mmHg, or reporting to have used antihypertensive medication during the past two weeks or having hypertension during the past year [7]. Standardized BP measurements were obtained by trained nurses during a home visit as part of the BHES fieldwork, using an electronic tensiometer (type Omron M6) [27]. Respondent’s SBP and DBP were determined by taking the respective averages of the last two out of three BP measurements [27].

The proportions of the hypertensive population reaching the different stages of the CoC were estimated using the following definitions. Being *screened* for hypertension was defined as having had a BP measurement less than 5 years ago [7, 31]. This cut-off was chosen as guidelines recommend that BP should be measured at least every 5 years, and more frequently when opportunities arise [31]. Being *diagnosed* was defined as self-reported hypertension in the past year [7]. Being *linked to care* was defined as being followed by a health care professional for hypertension in the past year. Being in *treatment* was defined as either self-reported use of medication or following a diet to treat hypertension in the past year [22, 29]. As the cardiovascular risk of the hypertensive patient needs to be reassessed annually [29, 32], including the determination of cholesterol levels, we defined being *followed up* as having had a blood cholesterol level measurement in the past year. Finally, being *controlled* for hypertension was defined as being treated for hypertension and currently having SBP < 140 mmHg and DBP < 90 mmHg [7, 22].

### Potential determinants of hypertension care

Socio-demographic factors included *age*, *sex* (male [ref.], female), *marital status* (married/cohabiting [ref.], single, divorced/widow(er)) and *educational level*. The latter is recoded into three categories: low (lower secondary education or lower) [ref.], middle (higher secondary education) and high (higher education).

In line with similar studies [22–25], we also included body mass index (BMI) and current smoking status as potential determinants of hypertension care. *BMI* is measured as kg/m² based on self-reported weight and height and included as a continuous predictor. *Smoking status* is a dummy variable indicating whether the respondent is a current smoker (i.e. having smoked at least 100 cigarettes in a lifetime and reporting to currently smoke ‘daily’ or ‘occasionally’) or not.

Apart from sociodemographic and lifestyle characteristics, we also included several variables that are presumed to be related to hypertension care and outcomes based on previous literature. First, as Belgium performs poorly in ensuring the financial accessibility of healthcare to the least well-off — with levels of unmet medical care for the lowest income quantile as high as 5.6–6.7% [16, 17] — we included a categorical predictor *perceived financial hardship.* It was measured using the survey question: Thinking of your household’s total available income, is your household able to make ends meet?”. Answers ranged on a 6-point Likert scale and were recoded into three categories: high (‘with great difficulty’ and ‘with difficulty’), moderate (‘with some difficulty ‘fairly easily’) and none (‘easily’ and ‘very easily’).

Poor health literacy may be a potential cognitive barrier to optimal hypertension care, as recent meta-analyses showed that it was associated with increased non-adherence to treatment recommendations among patients with chronic diseases [33, 34]. Respondents’ *health literacy* was assessed using the shortened 6-item version of the European Health Literacy Survey Questionnaire (HLS-EU-Q6) [35] and included as a continuous predictor ranging between 1 and 4, with higher scores indicating higher levels of health literacy.

Numerous studies reported a positive association between depression and uncontrolled hypertension [36, 37]. Apart from sharing common pathophysiological pathways, thereby potentially negatively affecting one another [36], depression and anxiety may lead to lower treatment adherence [37, 38] by reducing one’s perceived self-efficacy [39] and impairing one’s interest in and cognitive ability to follow treatment recommendations [40]. We included *psychological distress* as a continuous predictor, measured using the 12-item version of the General Health Questionnaire (GHQ-12) [41]. The scale resulted in a score ranging between 0 and 12, with higher scores indicating higher levels of psychological distress.

Finally, previous studies have shown a higher overall quality of care for patients with multiple chronic conditions [42–45], in particular among patients with concordant conditions [43, 45]. Hence, we expect that comorbidity is associated with an increased likelihood of progressing through the continuum of hypertension care. In the current analysis, *comorbidity* was included as a dummy variable indicating whether the hypertensive patient self-reported to have at least one co-occurring chronic condition (out of a total of 24 conditions) in the past 12 months [46, 47]. The full list of chronic conditions is provided in the text A1 (supplementary data).

### Statistical analysis

The analysis consisted of two steps and was performed in R (version 4.2.0) [48]. The stratified multistage clustered survey design was accounted for at both steps using the Survey package [49], resulting in estimates that are representative at the level of the Belgian population.

**Fig. 1:**
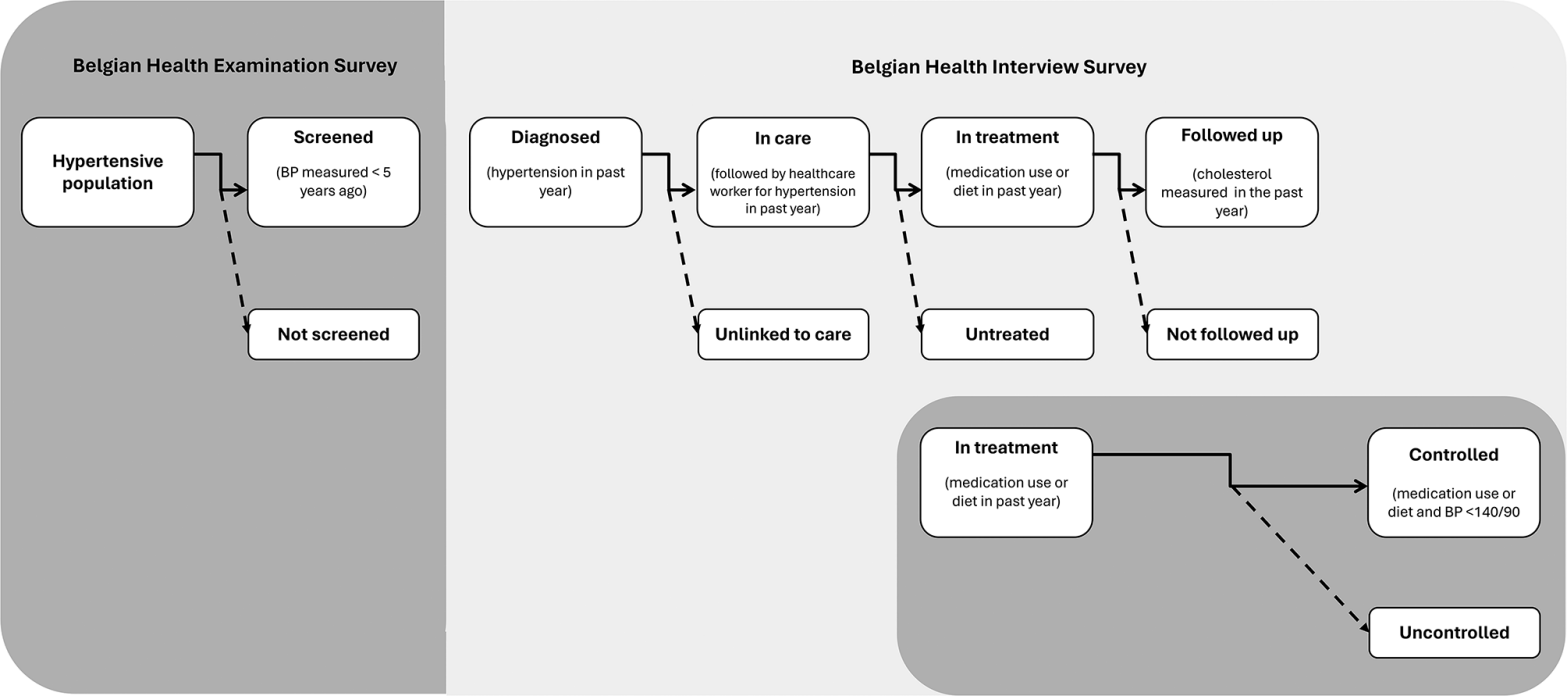
Flowchart of the stages of the cascade of hypertension care

First, we followed a CoC approach to identify points of greatest attrition along the hypertension care trajectory [18, 19]. The proportions for each cascade step were estimated using a fixed denominator (i.e., the number of hypertensive individuals aged 40–79 years). As our data comes from two sources, it does not allow to follow the same set of individuals across all stages of the CoC^1^, and hence, to estimate the proportion reaching any given stage conditional on having reached *all* previous stages. This resulted in a hybrid approach (see Fig. 1): for consecutive cascade stages based on a single data source, the proportions reaching a particular stage are estimated conditional on having reached the previous stage; for the remaining bars, the proportions are estimated following an unconditional approach. Participants with missing information on the cascade stages (12 of the BHIS and 8 of the BHES sample) were excluded from the CoC analysis. A more detailed elaboration on the operationalization of each bar is summarized in Table A1 (supplementary data).

Second, high-risk groups for attrition from hypertension care were identified by quantifying the drops in the cascade as dependent variables and analysing its associations with several predictor variables. Due to the small sample size of the BHES, this was only assessed for the conditional cascade stages based on the BHIS. Hence, the associations with three outcome variables were studied (see Fig. 1): (1) unlinked vs. linked to care (among diagnosed individuals, sample 1); (2) untreated vs. treated (among those diagnosed with hypertension and linked to care, sample 2); and (3) not followed up vs. followed up (among those diagnosed with hypertension, linked to care and following a treatment, sample 3). Both bivariate and multivariate Cox regression models with equal follow-up times and robust variances [50] were fitted, yielding interpretation of the exponentiated parameter estimates in terms of prevalence ratios (PR). Statistical significance was considered in case of a p-value of < 0.05.

Because of the high proportion of observations with a missing value on at least one variable—in the first sample for instance, this amounts to 30.3% —missing values were multiple imputed by chained equations using the MICE package [51] prior to analysis. The imputation process generated 100 datasets for each of the three samples and was informed by all variables included in the analysis and several additional auxiliary variables (birth country, employment status, equivalent household income in quantiles and an indicator of polypharmacy). The Cox regression models were subsequently fit to each of the imputed datasets and the resulting parameter estimates were pooled according to Rubin’s rules [52].

## Results

### The cascade of hypertension care

**Fig. 2:**
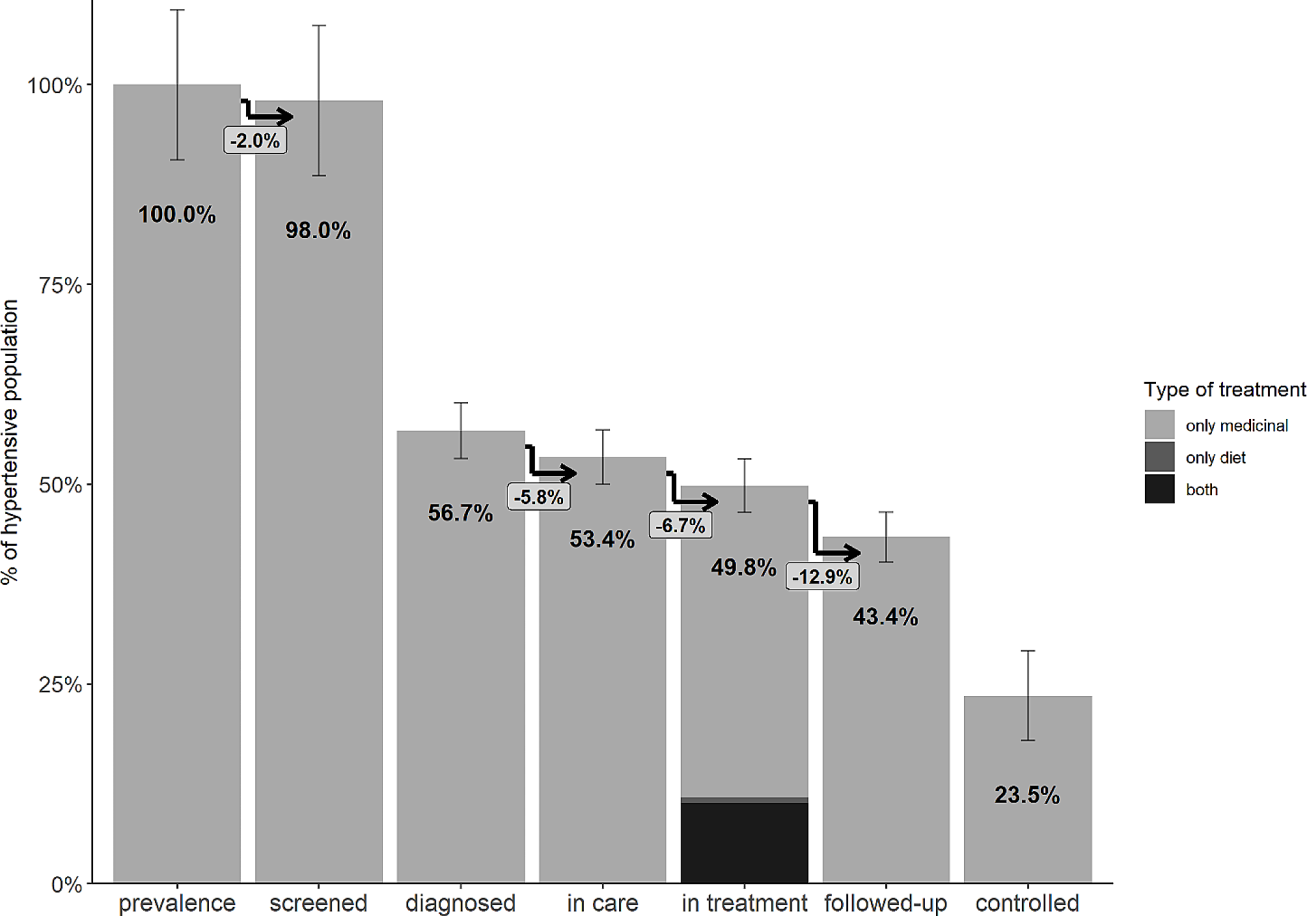
The cascade of hypertension care for the Belgian population aged 40–79 years

Descriptive characteristics for both the BHIS and BHES sample are provided in Table 1. Figure 2 presents the Belgian cascade of hypertension care. Each bar indicates the proportion of the hypertensive population reaching that particular cascade stage. The bars indicated with arrows are conditional on having reached the previous stage. The values in the boxes represent the conditional probabilities of attrition at the respective cascade stages. The remaining bars are estimated following the unconditional approach. The whiskers represent 95% confidence intervals.

**Table 1:** Weighted descriptive statistics for the BHIS and BHES participants aged 40–79 years (including those with and without hypertension)

	BHIS sample (*n* = 5932)		BHES sample (*n* = 813)	
	Value	% Missing^a^	Value	% Missing^a^
**Age**, mean (SD)	57.24 (10.79)		57.53 (11.41)	
**Gender**, *n* (%)		0.0		0.0
Female	3027 (51.1)		418 (51.9)	
Male	2905 (48.9)		395 (48.1)	
**Marital status**, *n* (%)		0.0		0.0
Married/cohabiting	3820 (65.9)		565 (69.1)	
Single	884 (13.1)		109 (12.1)	
Divorced/widow	1228 (21.0)		139 (18.8)	
**Education level**, *n* (%)		3.3		2.7
Low	1631 (27.1)		186 (24.4)	
Medium	1818 (33.6)		272 (36.5)	
High	2290 (39.4)		333 (39.1)	
**Financial hardship**, ***n (%)***		2.0		0.6
None	2505 (45.7)		374 (48.5)	
Moderate	2339 (38.6)		305 (38.1)	
High	968 (15.7)		129 (13.4)	
**Health literacy, mean (SD)**	3.13 (0.60)	22.1	3.10 (0.58)	11.7
**Psychological distress, mean (SD)**	1.65 (2.77)	15.6	1.66 (2.83)	5.9
**BMI, mean (SD)**	26.47 (4.97)	1.4	26.56 (4.83)	0.9
**Smoker**, ***n (%)***		16.0		6.9
No	4016 (80.6)		603 (82.5)	
Yes	966 (19.4)		154 (17.5)	
**Comorbidity**^b^, ***n (%)***		0.2		0
No	4827 (81.3)		685 (83.4)	
Yes	1096 (18.7)		128 (16.6)	

BHIS = Belgian Health Interview Survey; BHES = Belgian Health Examination Survey; SD = standard deviation

^a^Proportion missing values in the sample before multiple imputation

^b^Comorbidity is defined as self-reporting to have at least one co-occurring chronic condition (out of a total of 24 conditions) in addition to hypertension

The estimated prevalence of hypertension among the Belgian population aged 40–79 years was 43.3% (95% CI: 39.2–47.3). Of these, 98% had their BP measured in the past 5 years, but only slightly more than half (56.7%) were diagnosed with hypertension. Among those diagnosed, 5.8% reported that they were not followed by a healthcare professional for hypertension, resulting in 53.4% of the hypertensive population being linked to care. Among those who were diagnosed and linked to care, 6.7% remained untreated. Hence, about half (49.8%) the hypertensive population was in treatment, with the majority taking antihypertensive medication only (39%) and 10.1% both using medication and following a diet. A small minority (0.7%) followed a diet without taking antihypertensives. Among those who were diagnosed, linked to care and following treatment, 12.9% reported that they had no blood cholesterol measurement in the past year, resulting in 43.4% of the hypertensive population being followed up appropriately. Finally, only about a quarter of the hypertensive population (23.5%) was well controlled.

### Determinants of hypertension care

Table 2 presents the results of the bivariate and multivariate Cox regression models with the selected drops in the cascade as outcome variables. Descriptive statistics for each of the samples can be found in Table A2 (supplementary data). Among those diagnosed with hypertension, males and widow(er)s or divorcees were almost twice as likely to be *unlinked to care* than women (adjusted prevalence ratio (APR): 1.81; 95% CI: 1.04–3.15) and married or cohabiting couples (APR: 1.92 95% CI: 1.06–3.45), respectively, net of all other variables in the model. Older age was associated with improved patient retention: the estimated likelihood of being unlinked to care was, net of all other variables, 6% lower for each additional year of age (95% CI: 0.92–0.97). Smokers (PR: 2.56; 95% CI: 1.35–4.85) and those without comorbid conditions (1/PR: 1.89; 95% CI: 1.03–3.45) were significantly more likely to be unlinked to care than their respective counterparts, but these relationships were no longer significant after adjusting for other variables.

**Table 2:** Results of the cox regression models with the following drops in the cascade as outcome variables: not linked to care, untreated and not followed up

	Sample 1^a^ (*n* = 1427)						Sample 2^b^ (*n* = 1332)						Sample 3^c^ (*n* = 1243)					
	Diagnosed but unlinked to care (1/0)						Linked to care but untreated (1/0)						in treatment but not followed up (1/0)					
	PR	CI-95%		APR	CI-95%		PR	CI-95%		APR	CI-95%		PR	CI-95%		APR	CI-95%	
**Age** (in years)	0.94	[0.92; 0.97]	***	0.94	[0.92; 0.97]	***	0.96	[0.93; 0.99]	**	0.96	[0.93; 0.99]	**	0.97	[0.96; 0.99]	**	0.97	[0.96; 0.99]	**
**Sex**																		
Female	ref.			ref.				ref.		ref.			ref.			ref.		
Male	1.91	[1.11; 3.29]	*	1.81	[1.04; 3.15]	*	0.95	[0.57; 1.58]		1.05	[0.66; 1.65]		0.65	[0.44; 0.97]	*	0.55	[0.37; 0.83]	**
**Marital status**																		
Married/cohabiting	ref.			ref.			ref.			ref.			ref.			ref.		
Single	1	[0.41; 2.46]		0.64	[0.28; 1.47]		0.89	[0.41; 1.93]		0.69	[0.31; 1.56]		1.34	[0.82; 2.18]		1.34	[0.84; 2.12]	
Divorced/widow(er)	1.44	[0.78; 2.63]		1.92	[1.06; 3.45]	*	1.01	[0.54; 1.87]		1	[0.55; 1.83]		0.83	[0.5; 1.38]		1.05	[0.63; 1.75]	
**Education level**																		
Low	ref.			ref.			ref.			ref.			ref.			ref.		
Middle	0.73	[0.37; 1.42]		0.52	[0.26; 1.03]		1.22	[0.69; 2.17]		0.92	[0.53; 1.59]		1.81	[1.08; 3.04]	*	1.59	[0.96; 2.64]	
High	0.95	[0.49; 1.85]		0.7	[0.36; 1.36]		0.55	[0.29; 1.05]		0.44	[0.23; 0.83]	*	1.56	[0.92; 2.65]		1.3	[0.73; 2.31]	
**Financial hardship**																		
None	ref.			ref.			ref.			ref.			ref.			ref.		
Moderate	1.07	[0.55; 2.05]		1.13	[0.59; 2.14]		2.35	[1.23; 4.46]	**	2.12	[1.16; 3.87]	*	0.78	[0.48; 1.24]		0.85	[0.54; 1.32]	
High	1.43	[0.68; 3.03]		1.16	[0.54; 2.51]		2.22	[1.09; 4.53]	*	1.59	[0.73; 3.46]		0.75	[0.42; 1.34]		0.9	[0.5; 1.62]	
**Health literacy**	0.89	[0.58; 1.37]		0.9	[0.59; 1.37]		1.23	[0.78; 1.95]		1.42	[0.92; 2.17]		1.17	[0.82; 1.68]		1.08	[0.75; 1.55]	
**Psychological distress**	1.04	[0.96; 1.13]		0.99	[0.9; 1.09]		1.11	[1.04; 1.19]	**	1.1	[1.02; 1.18]	*	0.97	[0.9; 1.04]		0.98	[0.91; 1.06]	
**BMI**	0.97	[0.92; 1.02]		0.95	[0.89; 1]		0.94	[0.88; 1.01]		0.93	[0.87; 1]	*	0.97	[0.93; 1.01]		0.98	[0.94; 1.02]	
**Smoker**																		
No	ref.			ref.			ref.			ref.			ref.			ref.		
Yes	2.56	[1.35; 4.85]	**	1.79	[0.93; 3.43]		1.79	[0.95; 3.35]		1.25	[0.66; 2.36]		0.71	[0.38; 1.3]		0.66	[0.35; 1.25]	
**Comorbidity** ^d^																		
No	ref.			ref.			ref.			ref.			ref.			ref.		
Yes	0.53	[0.29; 0.97]	*	0.66	[0.35; 1.23]		0.86	[0.5; 1.46]		0.75	[0.42; 1.33]		0.52	[0.33; 0.8]	**	0.56	[0.36; 0.88]	*

PR = prevalence ratio; APR = adjusted prevalence ratio (adjusted for all predictor variables listed); CI-95% = 95% confidence interval

^a^individuals diagnosed with hypertension

^b^individuals diagnosed with hypertension and linked to care

^c^individuals diagnosed with hypertension, linked to care and following a treatment

^d^Comorbidity is defined as self-reporting to have at least one co-occurring chronic condition (out of a total of 24 conditions) in addition to hypertension

*p < 0.05 ; **p < 0.01 ; ***p < 0.001

Among those diagnosed and enrolled in care for hypertension, those of older age and with a higher BMI were less likely to be *untreated*. Controlling for other factors, the probability of being untreated is respectively 4% (95% CI: 0.93–0.99) and 7% (95% CI: 0.87-1.00) lower for each additional year of age and one-unit increase in BMI. On the other hand, being untreated for hypertension was more prevalent among those experiencing higher levels of psychological distress (APR: 1.1; 95% CI: 1.02–1.18), those that are lower educated compared to higher educated (1/APR: 2.27; 1/95% CI: 1.20–4.35) and those experiencing moderate financial hardship compared to those without financial hardship (APR: 2.12; 95% CI: 1.16–3.87). Those experiencing high levels of financial hardship were also significantly more likely to be untreated (PR: 2.22; 95% CI: 1.09–4.53), but this significant relationship disappeared after controlling for other variables.

Finally, among those that were diagnosed, enrolled in care and following treatment for hypertension, *not being followed up* appropriately by means of a yearly blood cholesterol measurement was more than one and a half times as prevalent among women (1/APR: 1.81; 1/95% CI: 1.20–2.70) and those without comorbid conditions (1/APR: 1.79; 1/95% CI: 1.14–2.78) than among their respective counterparts, net of all other variables in the model. Each additional year of age was associated with a 3% reduction (95% CI: 0.96–0.99) in the likelihood of not being followed up, controlling for the other factors. Not receiving the appropriate follow-up was also significantly more prevalent among respondents with a medium rather than a low level of education (PR: 1.81; 95% CI: 1.08–3.04), but this relationship was no longer significant in the multivariate model.

For comparison, the analysis was also performed on the subset of cases with complete information. Similar results were obtained, although the estimates based on the multiple imputed datasets were generally more efficient, resulting in more statistically significant associations (see Table A3, supplementary data). Moreover, due to the conditional nature of the CoC, the associations with the three different outcome variables are assessed on different and increasingly selective subsamples. To examine whether this induced selectivity has impacted the results, we also performed the analysis for the three outcome variables on the same subsample of diagnosed individuals (sample 1). The results did not differ substantially (see Table A4, supplementary data).

## Discussion

The aim of the current study was to estimate the cascade of hypertension care for the Belgian population aged 40–79 and to assess patient characteristics associated with attrition at various stages of hypertension management. We found that patients are lost at each stage of the cascade and only about one in four hypertensive patients were well controlled (23.5%). The largest loss occurred early on in the cascade: while 98% of the hypertensive population were screened for hypertension, only 56.7% were aware of their condition through a diagnosis. Once diagnosed, the Belgian health system retains patients fairly well along the continuum of care: 53.4% of the hypertensive study population were linked to care, 49.8% were in treatment and 43.4% were followed up by means of a blood cholesterol measurement in the past year. Nevertheless, the results of the current study show substantial differences between population sub-groups and shed light on some high-risk profiles for being unlinked to hypertension care, untreated or not followed up adequately.

Only slightly more than half (56.7%) of the hypertensive study population were aware of their condition. This proportion is lower than the awareness rates found among similar aged populations in all but one of the twelve high-income countries studied by Zhou et al. [4]. Not surprisingly, almost all of these countries with the highest awareness rates — such as Canada, Germany, the USA and South Korea — have national programmes for hypertension screening and education in place [4]. Perhaps the adoption of a similar program in Belgium may also improve awareness rates, and in turn, the proportion with controlled hypertension.

Nevertheless, this large proportion of undetected hypertension is somewhat surprising, given that almost everyone (98%) of the hypertensive population were screened by means of a BP measurement in the past 5 years. Previous research has shown, however, that diagnostic inertia — i.e. when physicians observe elevated BP measurements but label the respective patients as normotensive [53] — occurs as frequently as in 32.5% of the hypertensive cases [53], which may therefore partly account for the large observed loss of patients between the screening and diagnosis stage.

The factors contributing to such a conservative attitude of physicians when interpreting BP readings are multiple and often induced by uncertainty regarding the reliability of BP measurements [54]. These uncertainties can, for instance, arise from the use of invalidated or incorrectly calibrated measurement instruments, high individual variability in BP or suspected white coat hypertension [54]. The coexistence of different, and sometimes inconsistent, (inter)national guidelines on the management of hypertension may also cast doubt and confusion among physicians, potentially leading to inaction [54–56]. Moreover, physicians may refrain from formally diagnosing hypertension based on good clinical reasoning [55], which is especially common among the elderly and those with comorbid conditions [54].

Adequate interventions are needed to reduce the rate of undiagnosed hypertension. The use of 24-hour ambulatory BP monitoring (ABPM) may overcome many of the diagnostic uncertainties outlined above. 24-hour ABPM is currently considered as the state-of-the-art method for diagnosing hypertension [57–59]. By providing numerous objective BP readings over the course of 24 hours in the patient’s usual setting, the readings are not influenced by the white-coat effect, allow to detect masked and nighttime hypertension, and provide insight into BP variability [57–59]. Yet, despite these clear benefits, and although ABPM has been shown to be a cost-effective strategy for detecting hypertension [57], it is currently not reimbursed by the Belgian health insurance [57, 59] and not recommended in the Belgian guidelines for the management of hypertension in primary care [29], hampering its widespread adoption. Apart from promoting the use of 24-hour ABPM, electronic health records (EHRs) that include data on BP readings can be used in an algorithm-based approach to identify hypertensive cases that might have been missed otherwise [60, 61]. In a study in the United States, for instance, 47% of those that were algorithmically identified as having undiagnosed hypertension and had subsequently their BP measured, were found to have hypertension [60].

It is important to note, however, that the high rate of undiagnosed hypertension found in the current study may, at least to some extent, be due to methodological artefacts. In line with major epidemiological protocols [62, 63] all BP measurements were recorded during a single visit [27], whereas diagnostic guidelines recommend measuring BP on multiple occasions [29, 31]. This may have resulted in an overestimation of the prevalence [64], although relying on the last two out of three BP measurements most likely has attenuated this bias [64]. In contrast, the use of self-reported hypertension to identify those with known hypertension may have resulted in an underestimation of the true awareness rate due to recall bias and misunderstanding of the questions being asked [65]. The latter may be particularly the case in the present study, as respondents were merely asked whether they had a high BP during the past 12 months, rather than whether they had been diagnosed by a healthcare professional. We suspect that some hypertensive individuals with normalized BP because of treatment, may have reported that they do not have an elevated BP.

Both methodological limitations may have contributed to an overestimation of the problem of undiagnosed hypertension in the current study. Future research should adopt more rigorous measurements to assess hypertension prevalence and awareness in Belgium. For instance, using ABPM or BP measurements of multiple visits to identify those with undiagnosed hypertension, may lead to a more accurate description of the true magnitude of this problem in Belgium. Additionally, exploring characteristics that are associated with undiagnosed hypertension would enhance our understanding of this problem.

Once diagnosed, those with a higher risk for developing hypertensive end organ damage, such as those of older age, with a higher BMI or comorbid conditions, are generally better retained along the continuum of care. These findings are largely in line with observations made in other studies [23–25] and may in part reflect the Belgian healthcare system’s better performance in the field of curative rather than preventive care [16]. A greater perceived need to provide better quality care for elderly hypertensive patients with comorbid conditions [44], in addition to increased contact with healthcare services [42, 44] and use of specialist care [42], may all have contributed to this improved hypertension care.

Furthermore, our results show that treatment rates are lower among those experiencing psychological distress, which corroborates earlier findings of lower treatment adherence among those with poor mental health [37, 38]. This suggest that screening for depression and anxiety in hypertensive patients, and subsequently managing the symptoms thereof, may be a fruitful strategy to improve hypertension treatment and control rates. It should be noted, however, that part of the negative association between psychological distress and hypertension treatment may be explained by potential undesirable or dangerous interaction effects when simultaneously using certain antidepressants and antihypertensives [66], highlighting the need of searching for therapies that can be combined safely.

Our results also indicate that in a high-income country such as Belgium, with a compulsory health insurance system [16], treatment rates are lower among those experiencing financial hardship. This underscores earlier findings that Belgium performs poorly in ensuring the financial accessibility of healthcare to the least well-off [16, 17], and suggests that the out-of-pocket costs for antihypertensive medication in Belgium — which was estimated at 68.2 Euros per patient in the year 2012 [67] — may be too high for economically vulnerable groups. Indeed, numerous studies have shown that higher out-of-pocket costs for hypertension medication are associated with lower rates of treatment adherence [67, 68]. Continuous patient education on the importance of medication adherence to achieve BP control, as well as healthy living to prevent hypertension, remains necessary, with special attention for those of lower socio-economic status.

Finally, the results of the cascade indicate that despite receiving treatment, BP control remains often suboptimal. When considering patients who self-reported to be in treatment, only about half achieved BP control (23.5/49.8 = 47.2%). While it is difficult to draw direct comparisons due to the small sample used for estimation and the resulting wide 95% CIs, this finding is consistent with the BP control rates of 44–45% recently observed among treated hypertensive patients from Belgium and Luxembourg [8, 10]. Our estimate is, however, slightly higher than those found in earlier studies in Belgium [9, 11], which may be indicative of improvements over time.

Although our study does not provide insight into potential explanations for suboptimal BP control among treated patients, poor adherence to treatment [69, 70] and therapeutic inertia [69] are likely important contributing factors. In Belgium, 38.9% of treated hypertensive patients self-reported as being non-adherent to therapy [70], and a similar proportion of uncontrolled hypertensive patients were rated by their general practitioner (GP) as having insufficient treatment adherence [69]. Indications of therapeutic inertia are found in recent studies in primary care in Belgium and Luxembourg, showing that GPs tend to overestimate BP control rates [8] and leave treatment unchanged in 16% of uncontrolled hypertensive patients [69]. In addition, monotherapy is still widely used among uncontrolled hypertensive patients [69] and free-pill combinations remain the predominant mode of combination therapy in Belgium [8], despite the European guidelines for hypertension management recommending single-pill combination therapy at the initiation of treatment [31].

### Strengths and limitations

This study is the first study to provide recent evidence on the state of hypertension management in Belgium, based on nationally representative survey data, and along the entire continuum of hypertension care by using an extended CoC approach. By including information on the proportion screened, linked to care and followed-up, we improved upon the majority of previous studies that only assessed hypertension prevalence, awareness, treatment and control. The inclusion of information on the proportion screened, for instance, shed light on the fact that in Belgium, the large proportion of undetected hypertension is not simply due to physicians measuring BP infrequently, but rather seems a problem of diagnostic inertia.

Apart from the strengths, several limitations should be acknowledged. In addition to those already discussed, the major limitation of the current study is the use of cross-sectional rather than longitudinal data. As such, each cascade stage includes a different set of individuals who are unlikely to be exchangeable, which may give rise to false impressions of the efficacy of improvements early on in the cascade on the proportions at later stages [71]. For instance, those diagnosed with hypertension may differ from those with undiagnosed hypertension, so that improving hypertension detection may not yield similar increases in treatment and control rates as expected based on the cross-sectional cascade. Moreover, a cross-sectional cascade fails to capture the highly dynamic nature of hypertension care where patients not only move forward but also regress back to earlier stages in the cascade [72]. The cross-sectional nature of the data does also not allow for a causal interpretation of the regression results, as we analyse associations between levels of — rather than changes in — the predictor variables and attrition from hypertension care. Finally, the majority of data used to estimate the CoC concerns self-reported data. Due to recall bias, this may have resulted in an under- or overestimation of the proportions reaching the respective CoC stages. While validation studies have shown that self-reports of hypertension and contact with healthcare providers yield only a slight underestimation compared to medical record and administrative data [65, 73, 74], self-reports of screening services, such as BP and cholesterol measurements, have been shown to be less consistent, with a tendency of overreporting [73].

## Conclusion

The current study demonstrated the importance of using an extended CoC approach to evaluate the delivery of hypertension care in a high-income country such as Belgium. We found that only about one in four hypertensive patients were well-controlled. Undiagnosed hypertension emerged as the most significant barrier to BP control, with only slightly more than half of the hypertensive patients being aware of their condition. To improve hypertension control rates, Belgian policymakers should first and foremost develop health interventions to reduce diagnostic inertia and improve the timely and accurate diagnosis of hypertension. Potentially fruitful interventions in this regard, include promoting the use of 24-hour ABPM as a means for diagnosing hypertension, and levering EHRs to algorithmically identify hypertensive patients that have been missed in the past.

Once diagnosed, the Belgian healthcare system retains patients fairly well along the continuum of hypertension care, yet some population sub-groups have a significantly higher risk for attrition from hypertension care. These differences were most pronounced with respect to treatment rates. To further improve hypertension control and reduce inequities in CVD, targeted health interventions are needed tailored specifically to the needs of these high-risk groups.

### Electronic supplementary material

Below is the link to the electronic supplementary material.

Supplementary Material 1

## Data Availability

The data are not publicly available. Access can be requested at: https://www.sciensano.be/en/node/55737/health-interview-survey-microdata-request-procedure.

## Footnotes

1 Strictly speaking, the BHES sample provides individual-level linked data across all stages of the CoC. The sample size is, however, too small to get efficient estimates for each bar, especially at the later stages in the cascade due to attrition along the continuum of care.

## Abbreviations

BP: Blood pressure
CVD: Cardiovascular disease
HICs: High-income countries
CoC: Cascade of care
BHIS: Belgian health interview survey
BHES: Belgian health examination survey
SBP: Systolic blood pressure
DBP: Diastolic blood pressure
BMI: Body mass index
HLS-EU-Q6: 6-item version of the European health literacy survey questionnaire
GHQ-12: 12-item version of the General health questionnaire
PR: Prevalence ratio
APR: Adjusted prevalence ratio
95% CI: 95% Confidence interval
ABPM: Ambulatory blood pressure monitoring
EHRs: Electronic health records
GP: General practitioner
