# Supplementary material for: Unravelling the Belgian cascade of hypertension care and its determinants: insights from a cross-sectional analysis
Source: BMC Public Health. 2024 Jun 14;24:1559. doi: 10.1186/s12889-024-19010-x (PMC11177511; doi:10.1186/s12889-024-19010-x)
Supplement: Supplementary file 1 — Supplementary Material 1 [file 12889_2024_19010_MOESM1_ESM.pdf]

Supplementary data

Unravelling the Belgian cascade of hypertension care and its determinants: Insights from a cross-sectional analysis

Philippe Bos, Edwin Wouters, Katrien Danhieux, Josefien Van Olmen, Roy Remmen, Kerstin Klipstein-Grobusch, Daniel Boateng, Veerle Buffel

Table of contents

Text A1. Operationalization of the comorbidity indicator 2

Table A1. Numerators, denominators and data sources used to construct the cascade of hypertension care 2

Table A2. Descriptive statistics for each of the subsamples used in the regression analyses 3

Table A3. Results of the Cox regression models estimated on the subset of complete cases 4

Table A4. Results of the cox regression models estimated on the sample of diagnosed hypertensive individuals with the unconditional drops in the cascade as outcome variables 6

Text A1. Operationalization of the comorbidity indicator

As an indicator of *comorbidity*, we constructed a dichotomous variable indicating whether the respondent self-reported to have at least one co-occurring chronic condition in addition to hypertension, in the past 12 months preceding the survey. The list of comorbidities comprised the following 24 conditions: asthma, bronchitis, heart disease, rheumatoid arthrosis or arthritis, diabetes, cancer, stroke, allergy, stomach ulcer, cirrhosis of the liver, serious headache, depression, thyroid problems, glaucoma, cataract, Parkinson's disease, epilepsy, chronic fatigue, osteoporosis, chronic bowel disease, kidney disease, chronic cystitis, chronic or serious skin disease and gallstones.

Table A1. Numerators, denominators and data sources used to construct the cascade of hypertension care

Cascade bars	Numerator	Denominator	Data
1. Prevalence	$\hat{t}_{(hypertension)}$	$\hat{t}$	BHES
2. Screened	$\hat{t}_{(screened,\ hypertension)}$	$\hat{t}_{(hypertension)}$	BHES
3. Diagnosed	$\hat{t}_{(diagnosed)}$	$\hat{t}_{(hypertension)}$	BHIS
4. Linked to care	$\hat{t}_{(linked\ to\ care, diagnosed)}$	$\hat{t}_{(hypertension)}$	BHIS
5. In treatment	$\hat{t}_{(in\ treatment, linked\ to\ care, diagnosed)}$	$\hat{t}_{(hypertension)}$	BHIS
6. Followed-up	$\hat{t}_{(followed-up\ , in\ treatment\ , linked\ to\ care, diagnosed)}$	$\hat{t}_{(hypertension)}$	BHIS
7. Controlled	$\hat{t}_{(controlled, in\ treatment)}$	$\hat{t}_{(hypertension)}$	BHES

The population was restricted to individuals aged 40-79 years; $\hat{t}$ denotes the estimated population total; $\hat{t}_{(i)}$ indicates the estimated population total for condition *i;* The Belgian Health Interview Survey is abbreviated as BHIS, whereas the Belgian Health Examination Survey is abbreviated as BHES.

Table A2. Descriptive statistics for each of the subsamples used in the regression analyses

	Sample 1[a]		Sample 2[b]		Sample 3[c]	
	n= 1427		n = 1332		n = 1243	
	n (%)/ mean (SD)[d]	% missing[e]	n (%)/ mean (SD)[d]	% missing[e]	% / mean (SD)[d]	% missing[e]
**Age** (in years)	61.75 (10.29)	0.0	62.13 (10.17)	0.0	62.43 (10.01)	0.0
**Gender**		0.0		0.0		0.0
Female	731 (51.6%)		695 (52.6%)		642 (52.5%)	
Male	696 (48.4%)		637 (47.4%)		601 (47.5%)	
**Marital status**		0.0		0.0		0.0
Married/cohabiting	340 (65.6%)		313 (66.0%)		292 (66.0%)	
Single	896 (10.5%)		840 (10.5%)		784 (10.6%)	
Divorced/widow	191 (23.9%)		179 (23.5%)		167 (23.5%)	
**Education level**		3.6		3.4		3.2
Low	476 (33.3%)		439 (33.0%)		407 (32.9%)	
Medium	435 (33.4%)		409 (33.7%)		376 (32.9%)	
High	465 (33.4%)		439 (33.2%)		420 (34.2%)	
**Financial hardship**		2.3		2.0		2.2
None	268 (40.8%)		246 (41.0%)		223 (42.4%)	
Moderate	598 (40.7%)		561 (40.7%)		514 (39.8%)	
High	528 (18.6%)		498 (18.2%)		479 (17.9%)	
**Health literacy**	3.05 (0.63)	23.5	3.05 (0.63)	22.7	3.05 (0.63)	23.3
**Psychological distress**	1.86 (2.91)	14.2	1.82 (2.90)	13.2	1.74 (2.80)	13.1
**BMI**	28.49 (5.25)	1.5	28.55 (5.28)	1.7	28.65 (5.24)	1.6
**Smoker**		15.1		14.0		14.2
No	1004 (82.6%)		959 (83.7%)		899 (84.4%)	
Yes	208 (17.4%)		186 (16.3%)		168 (15.6%)	
**Comorbidity**[f]		0.4		0.4		0.3
No	326 (23.8%)		302 (23.0%)		276 (22.7%)	
Yes	1096 (76.2%)		1025 (77.0%)		963 (77.3%)	

[a] individuals diagnosed with hypertension
[b] individuals diagnosed with hypertension and linked to care
[c] individuals diagnosed with hypertension, linked to care and following a treatment
[d] For continuous variables, the weighted mean and corresponding standard deviation (SD) are shown; for categorical variables, the sample frequencies and weighted proportions are presented.
[e] Proportion missing values in the sample before multiple imputation.
[f] Comorbidity is defined as self-reporting to have at least one co-occurring chronic condition (out of a total of 24 conditions) in addition to hypertension.

Table A3. Results of the Cox regression models estimated on the subset of complete cases

	Sample 1[a]						Sample 2[b]						Sample 3[c]					
	diagnosed but unlinked to care (1/0)						linked to care but untreated (1/0)						in treatment but not followed up (1/0)					
	PR	CI-95%		APR	CI-95%		PR	CI-95%		APR	CI-95%		PR	CI-95%		APR	CI-95%	
**Age** (in years)	0.93	[0.9; 0.96]	***	0.93	[0.89; 0.96]	***	0.97	[0.94; 1]		0.97	[0.93; 1]		0.98	[0.96; 1]		0.98	[0.96; 1.01]	
**Sex**																		
Female	ref.			ref.				ref.		ref.			ref.			ref.		
Male	1.77	[0.86; 3.63]		1.47	[0.73; 2.97]		0.83	[0.47; 1.48]		1.04	[0.63; 1.74]		0.66	[0.41; 1.04]		0.62	[0.39; 0.98]	*
**Marital status**																		
Married/cohabiting	ref.			ref.				ref.		ref.			ref.			ref.		
Single	1.1	[0.31; 3.88]		0.63	[0.23; 1.72]		0.45	[0.17; 1.21]		0.41	[0.15; 1.08]		1.14	[0.63; 2.05]		1.09	[0.62; 1.92]	
Divorced/widow(er)	1.68	[0.77; 3.7]		2.65	[1.24; 5.69]	*	0.97	[0.48; 1.98]		0.94	[0.47; 1.9]		0.76	[0.41; 1.43]		0.87	[0.46; 1.67]	
**Education level**																		
Low	ref.			ref.				ref.		ref.			ref.			ref.		
Middle	1.23	[0.46; 3.31]		0.94	[0.37; 2.39]		1.08	[0.57; 2.07]		0.82	[0.46; 1.47]		1.61	[0.86; 3.02]		1.44	[0.75; 2.75]	
High	1.53	[0.58; 4]		1.2	[0.48; 3]		0.46	[0.23; 0.92]	*	0.38	[0.19; 0.74]	**	1.56	[0.83; 2.96]		1.36	[0.69; 2.68]	
**Financial hardship**																		
None	ref.			ref.				ref.		ref.			ref.			ref.		
Moderate	0.7	[0.31; 1.57]		0.74	[0.33; 1.65]		2.18	[1.09; 4.38]	*	1.97	[1.04; 3.73]	*	0.89	[0.5; 1.56]		1	[0.58; 1.72]	
High	1.34	[0.51; 3.48]		1.17	[0.46; 2.97]		1.83	[0.8; 4.15]		1.6	[0.68; 3.79]		1.07	[0.55; 2.09]		1.38	[0.71; 2.7]	
**Health literacy**	0.84	[0.56; 1.26]		0.81	[0.55; 1.2]		1.18	[0.73; 1.9]		1.35	[0.9; 2.04]		1.19	[0.84; 1.7]		1.15	[0.79; 1.69]	
**Psychological distress**	1.06	[0.97; 1.17]		0.99	[0.9; 1.09]		1.09	[1.01; 1.19]	*	1.08	[1; 1.17]	*	1	[0.93; 1.07]		1	[0.93; 1.08]	
**BMI**	0.99	[0.92; 1.07]		0.97	[0.89; 1.06]		0.92	[0.86; 0.99]	*	0.91	[0.84; 0.98]	*	0.95	[0.9; 1]	*	0.95	[0.9; 1]	*

**Smoker**														
No	ref.			ref.				ref.	ref.		ref.		ref.	
Yes	3.12	[1.49; 6.52]	***	2.17	[1.09; 4.3]	*	1.73	[0.87; 3.44]	1.25	[0.65; 2.38]	0.66	[0.31; 1.38]	0.6	[0.28; 1.3]
**Comorbidity**[d]														
No	ref.			ref.				ref.	ref.		ref.		ref.	
Yes	0.42	[0.2; 0.88]	*	0.56	[0.26; 1.2]		0.84	[0.47; 1.52]	0.72	[0.38; 1.37]	0.66	[0.4; 1.1]	0.67	[0.4; 1.13]

PR = prevalence ratio; APR = adjusted prevalence ratio (adjusted for all predictor variables listed); CI-95% = 95% confidence interval;
[a] individuals diagnosed with hypertension
[b] individuals diagnosed with hypertension and linked to care
[c] individuals diagnosed with hypertension, linked to care and following a treatment
[d] Comorbidity is defined as self-reporting to have at least one co-occurring chronic condition (out of a total of 24 conditions) in addition to hypertension.
* p<0.05 ; ** p<0.01 ; *** p<0.001

Table A4. Results of the cox regression models estimated on the sample of diagnosed hypertensive individuals with the unconditional drops in the cascade as outcome variables

	Sample 1[a]																	
	diagnosed but unlinked to care (1/0)						diagnosed but untreated (1/0)						diagnosed but not followed up (1/0)					
	PR	CI-95%		APR	CI-95%		PR	CI-95%		APR	CI-95%		PR	CI-95%		APR	CI-95%	
**Age** (in years)	0.94	[0.92; 0.97]	***	0.94	[0.92; 0.97]	***	0.95	[0.93; 0.98]	***	0.96	[0.93; 0.98]	***	0.97	[0.95; 0.98]	***	0.97	[0.96; 0.99]	**
**Sex**																		
Female	ref.			ref.			ref.			ref.			ref.			ref.		
Male	1.91	[1.11; 3.29]	*	1.81	[1.04; 3.16]	*	1.3	[0.86; 1.98]		1.28	[0.87; 1.88]		0.77	[0.55; 1.09]		0.65	[0.46; 0.93]	*
**Marital status**																		
Married/cohabiting	ref.			ref.			ref.			ref.			ref.			ref.		
Single	1	[0.41; 2.46]		0.64	[0.28; 1.46]		1.19	[0.64; 2.21]		0.84	[0.45; 1.55]		1.47	[0.96; 2.24]		1.37	[0.91; 2.07]	
Divorced/widow(er)	1.44	[0.78; 2.63]		1.91	[1.06; 3.44]	*	1.08	[0.65; 1.81]		1.17	[0.7; 1.98]		0.9	[0.59; 1.37]		1.06	[0.69; 1.63]	
**Education level**																		
Low	ref.			ref.			ref.			ref.			ref.			ref.		
Middle	0.73	[0.37; 1.42]		0.52	[0.26; 1.03]		1.11	[0.67; 1.83]		0.84	[0.52; 1.36]		1.74	[1.13; 2.67]	*	1.5	[0.97; 2.32]	
High	0.95	[0.48; 1.85]		0.69	[0.35; 1.35]		0.79	[0.45; 1.39]		0.59	[0.35; 1.01]		1.44	[0.91; 2.27]		1.2	[0.73; 1.96]	
**Financial hardship**																		
None	ref.			ref.			ref.			ref.			ref.			ref.		
Moderate	1.06	[0.55; 2.03]		1.11	[0.59; 2.12]		1.57	[0.91; 2.68]		1.49	[0.9; 2.48]		0.86	[0.57; 1.32]		0.95	[0.63; 1.43]	
High	1.43	[0.68; 3]		1.16	[0.54; 2.48]		1.79	[1; 3.2]	*	1.24	[0.66; 2.35]		1.02	[0.64; 1.64]		1.1	[0.69; 1.76]	
**Health literacy**	0.91	[0.58; 1.42]		0.92	[0.59; 1.42]		1.1	[0.77; 1.56]		1.25	[0.89; 1.75]		1.19	[0.88; 1.6]		1.11	[0.82; 1.5]	
**Psychological distress**	1.04	[0.96; 1.13]		0.99	[0.9; 1.09]		1.11	[1.05; 1.17]	***	1.09	[1.02; 1.16]	**	0.98	[0.92; 1.04]		0.97	[0.91; 1.04]	

**BMI**	0.97	[0.92; 1.02]		0.95	[0.89; 1]		0.96	[0.91; 1.01]		0.95	[0.9; 1]	*	0.97	[0.93; 1]		0.97	[0.94; 1.01]	
**Smoker**																		
No	ref.			ref.				ref.		ref.			ref.			ref.		
Yes	2.58	[1.36; 4.88]	**	1.81	[0.95; 3.46]		2.12	[1.29; 3.48]	**	1.47	[0.87; 2.47]		1.22	[0.8; 1.87]		1.1	[0.72; 1.68]	
**Comorbidity** [b]																		
No	ref.			ref.				ref.		ref.			ref.			ref.		
Yes	0.53	[0.29; 0.97]	*	0.66	[0.35; 1.23]		0.74	[0.46; 1.19]		0.7	[0.43; 1.15]		0.58	[0.4; 0.85]	**	0.64	[0.43; 0.95]	*

PR = prevalence ratio; APR = adjusted prevalence ratio (adjusted for all predictor variables listed); CI-95% = 95% confidence interval;

[a] individuals diagnosed with hypertension

[b] Comorbidity is defined as self-reporting to have at least one co-occurring chronic condition (out of a total of 24 conditions) in addition to hypertension.

* $p<0.05$; ** $p<0.01$; *** $p<0.001$
